# Eloquent silence: developmental functions of Class I histone deacetylases

**DOI:** 10.1016/j.gde.2008.10.001

**Published:** 2008-10

**Authors:** Vincent T Cunliffe

**Affiliations:** MRC Centre for Developmental and Biomedical Genetics and Department of Biomedical Science, University of Sheffield, Firth Court, Western Bank, Sheffield S10 2TN, United Kingdom

## Abstract

Histone deacetylases (HDACs) are essential catalytic components of the transcription silencing machinery and they play important roles in the programming of multicellular development. HDACs are present within multisubunit protein complexes, other components of which govern HDAC target gene specificity by controlling interactions with sequence-specific DNA-binding proteins. Here, I review the different developmental roles of the Sin3, NuRD, CoREST and NCoR/SMRT Class I HDAC complexes. With their distinct subunit composition, these versatile molecular devices function in many different settings, to promote axis specification and tissue patterning, to maintain stem cell pluripotency, facilitate self-renewal, guide lineage commitment and drive cell differentiation.

## Introduction

The machinery responsible for determining the transcriptional status of genes during development includes proteins that covalently modify core histones by acetylation or methylation of amino acids in their N-terminal domains. Acetylated histones recruit bromodomain transcription activators, whilst methylated histones recruit a variety of chromatin regulators, including chromodomain and PHD domain proteins. Acetylation marks are removed by histone deacetylases (HDACs), whereas histone demethylases are responsible for removing methyl marks from histones. HDACs can be grouped into structurally distinct Groups I, II, III and IV [1^•^]. This review focuses on the known developmental functions of the closely related Class I HDACs, HDAC1, HDAC2 and HDAC3, all of which are related to the *Saccharomyces cerevisiae* HDAC Rpd3. HDAC8 is an additional Class I member, but its roles in development remain to be elucidated.

In all model organisms studied, Class I HDACs are expressed during most stages of development and in many different cell types. Thus, their functions in different developmental contexts depend on their recruitment into different protein complexes. Amongst the Metazoa, Class I HDACs are found primarily in four distinct multiprotein complexes, known as the Sin3, NuRD, CoREST and NCoR/SMRT complexes, each of which is recruited to its target genes by different DNA-binding proteins. The known subunit composition of these complexes in *Caenorhabditis elegans*, *Drosophila* and vertebrates is summarised in Table 1. Class I HDACs are also found in more specialised complexes with specific DNA-binding proteins, such as CSL and TCF factors. Many of the components of Class I-HDAC-containing complexes have been highly conserved during animal evolution. The aim of this review is to summarise recent advances in understanding the diverse developmental roles of Class I HDACs as components of distinct protein complexes.

## Sin3 complex

The Sin3 complex comprises HDAC1, HDAC2, RbAp48, Sin3A/Sin3B, SAP18 and SAP30. Targets of this complex in mammalian cells include genes of the TGF-β/BMP signalling pathway and this HDAC-containing complex is brought to bear on TGF-β/BMP signalling through interactions with Smad proteins, which can either activate or repress target genes. Thus, in repressive mode, BMP signalling causes phosphorylation of Smad1, which promotes its association with Smad4 in mouse chondrocytes. In the nucleus, this complex binds to the Nkx3.2 transcription factor and stabilises its association with the Sin3–HDAC1 complex, leading to the BMP-induced transcriptional repression of Nkx3.2 targets [2].

**Figure 1:**
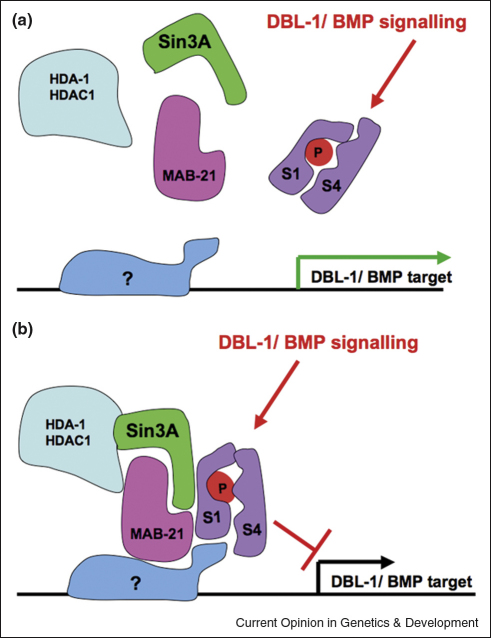
A possible model for DBL-1/BMP-4-induced transcriptional repression. **(a)** BMP signalling leads to the phosphorylation of Smad1, which complexes with Smad4. **(b)** Phospho-Smad1–Smad4 dimers bind to MAB-21 in the nucleus and this complex mediates the recruitment of Sin3A/HDAC1 to DNA-bound transcription factors at target genes, resulting in gene repression.

A similar scenario may occur in *C. elegans*, where the formation of the male sensory rays is dependent on the BMP signal DBL-1, which acts upstream of *mab-21*, a known genetic interactor of *sin-3* [3]. Interestingly, functional analysis and gene expression studies of the vertebrate MAB21 homologue, Mab21L2, implicate this gene in the development of the CNS and dorsal mesoderm. Mab21L2 interacts with the BMP4 effector Smad1, it has transcriptional repressor activity, and antagonises the ventralising effects of BMP pathway activity *in vivo* [4]. These observations suggest that in both worms and vertebrates, BMP-dependent transcriptional repression may be mediated by Smad-regulated, Mab21-dependent recruitment of Sin3-associated Class I HDACs to BMP-target genes (Figure 1).

In *Drosophila*, Rpd3/HDAC1 is recruited to segmentation genes by Bicoid and Pair-rule transcription factors. Repression of *hunchback* transcription in the head region by Bicoid is strictly dependent on the SIN3 component SAP18, and maternal-zygotic *sap18* mutant embryos exhibit severe head defects as well as segmentation abnormalities [5]. Recessive mutations in Rpd3/hdac1 are also embryonic lethal when homozygous and maternal-zygotic Rpd3 mutant embryos exhibit a pair-rule segmentation phenotype that is characterised by the variable loss of stripes of engrailed expression in even-numbered segments, consistent with a loss of Eve function [6]. Interestingly, the chick homologue of the pair-rule transcription factor Hairy, Hairy1, interacts with the Sin3 complex through direct binding to SAP18 [7]. Consistent with this finding, all three components of the Hairy1–Sin3–SAP18 complex are expressed in both presomitic mesoderm and newly formed somites, suggesting that the Sin3 complex represses Hairy1 target genes during segmentation of the paraxial mesoderm. Downstream of segmentation, an additional role for the *Drosophila* Sin3-associated Rpd3 in the specification of segmental identity was revealed by experiments demonstrating that the *Fab-7 cis*-regulatory element in the Bithorax complex represses the homeotic gene *Abd-B* through interactions between the Sin3–SAP18–Rpd3 complex and the *Fab-7*-bound GAGA factor [8]. Taken together, these studies suggest that Sin3 complexes are involved both in programming gene expression that is dependent on intercellular signalling activities and in maintaining long-term cell fate decisions which are executed downstream or independently of such signals.

## Nucleosome remodelling and deacetylase (NuRD) complex

The core components of the NuRD complex are HDAC1, HDAC2, RbAp46, RbAp48, the nucleosome remodelling ATP-ase Mi-2 (CHD-3/4), MBD3 and the SANT-domain-containing proteins MTA-1/2 [1^•^]. Many of these subunits are highly conserved between species and functional studies reveal the roles for NuRD in regulating cell fate in a wide range of model organisms.

**Figure 2:**
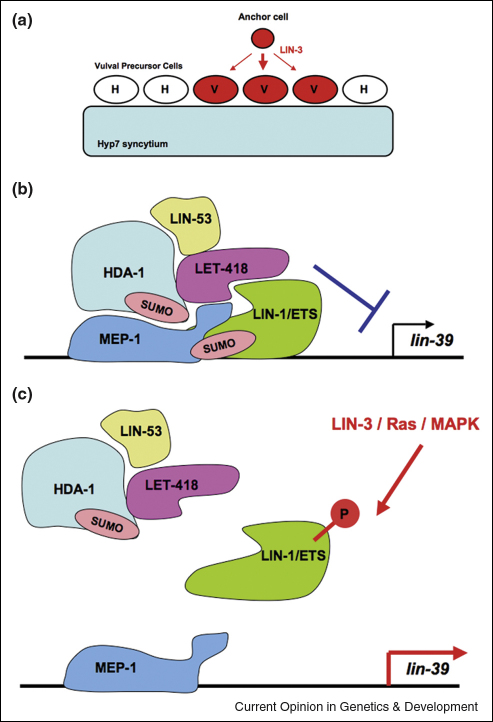
Role of the NuRD co-repressor complex in vulval fate specification. **(a)** Vulval fate (V) is induced in a limited number of VPCs by the short-range signal LIN3/EGF, secreted from the anchor cell (AC). VPCs that do not receive the LIN-3/EGF signal follow a Hypodermal fate (H) (see Ref. [9]). **(b)** A model for how NuRD antagonises vulval fate. NuRD components are the members of the synMuv group of proteins that repress vulval genes, for example *lin-39*. MEP-1 promotes the formation of a complex with NuRD and LIN-1/ETS on the *lin-39* regulatory elements. **(c)** EGF/RTK/Ras signalling results in LIN-1/ETS phosphorylation which disrupts LIN-1/ETS interactions with NuRD and MEP-1 and leads to target gene derepression.

In *C. elegans*, NuRD components have important functions in the regulation of vulval development. The phenotypes, interactions and molecular identities of an important class of vulval mutant genes, the synMuv class, indicate that the NuRD complex, along with other chromatin regulatory proteins, regulates the adoption of vulval fate by vulval precursor cells (VPCs) [9]. Vulval fate is induced in VPC by the binding of the anchor-cell (AC)-derived LIN-3/EGF signalling molecule to its receptor on the VPCs that lie close to the AC. Binding of LIN-3/EGF to its receptor activates the EGF/RTK/Ras signalling pathway in VPCs, which causes phosphorylation of the LIN-1/ETS transcription factor and activation/derepression of vulval genes such as *lin-39* (Figure 2a). In several different synMuv mutant genotypes, the EGF/RTK/Ras pathway is activated in more than the normal number of VPC, thus leading to a Multivulva phenotype. This ectopic pathway activation is thought to occur, at least partly, via the derepression of LIN-1/ETS target genes such as *lin-39* in VPCs [9]. Remarkably, many synMuv mutations lie in genes encoding chromatin regulators, including core NuRD components such as HDAC-1 (*hda-1*), RbAp48 (*lin-53*) and Mi-2 (*let-418*, *chd-3*) [9–15]. Studies of these genes suggest that NuRD likely acts in VPC to repress targets such as *lin-39*, in collaboration with the LIN-1/ETS transcription factor [14,15].

Further molecular analysis in C. elegans has also identified other proteins that interact with NuRD in transcriptional repression in VPC, such as MEP-1, a zinc finger protein which binds to LET-418/Mi-2 and HDA-1/Hdac1 [16]. The importance of the MEP-1 DNA-binding protein in NuRD-mediated repression of LIN-3/EGF was revealed by the discovery that LIN-1 is sumoylated and that this modification promotes an interaction with MEP-1, thus stabilising NuRD activity on LIN-1 target promoters [17^••^] (Figure 2b). Intriguingly, HDAC-1 is sumoylated in *C. elegans* and both SUMO and the E2 SUMO ligase UBC9 are members of the synMuv group [18^••^]. Moreover, mammalian HDAC1 is also sumoylated, and sumoylation of the EGF/RTK/Ras-responsive ETS transcription factor Elk-1 confers a Class I-HDAC-dependent transcriptional repressor function to Elk-1 [19]. However, the phosphorylation of Elk-1 or LIN-1 disrupts the transcriptional repressor complex and derepresses cognate target genes (Figure 2c), which, for LIN-1, can account for the LIN-3-inducibility of vulval fate in VPC. Taken together, these parallel observations in worms and mammals suggest that Class I HDACs within the NuRD complex repress EGF/RTK/Ras target genes by a SUMO-regulated mechanism such that the target promoters remain poised for activation by EGF/RTK/Ras signalling.

In vertebrates, the NuRD components MTA-1 and MTA-2 harbour conserved protein motifs known as SANT domains, which are also found in the *C. elegans* proteins EGL-27 and LIN-40/EGR-1 [20–22], and the members of the Atrophin protein family [23]. The *Drosophila* orthologue of Atrophin binds to and promotes the activity of HDAC1 and HDAC2 via its SANT domains [24], but it is not known whether other NuRD components also associate with Atrophin. Nevertheless, like NuRD components in the *C. elegans* vulva, *Drosophila* Atrophin negatively regulates the EGF/RTK/Ras pathway to control cell fate in the eye and wing imaginal discs, in co-operation with the ETS protein Yan, at least in part, by repressing the EGF target gene (and Notch Ligand) *Delta* [25]. The derepression of *Delta* in *Atrophin* mutants also parallels the derepression of the *C. elegans* Notch ligand gene *lag-2* observed in *hda-1* and other synMuv mutants [13].

In zebrafish, both HDAC1 and Atrophin-2 are required for the development of multiple organs and tissues, including the CNS, optic and otic vesicles, pharyngeal arches, neural-crest-derived melanophores and pectoral fins [26–33]. Atrophin-2 interacts genetically with the FGF signalling pathway in the CNS, mesoderm and endoderm [33]. Hdac1 also functions in the CNS, where it antagonises the Notch and Wnt signalling pathways and promotes responsiveness to Hedgehog signalling, thus facilitating cell-cycle exit of neural progenitors and the specification of differentiated neurons and glia [26,27,29,30]. The similarities between the *atrophin-2* and the *hdac1* mutant phenotypes suggest that they may be components of the same complexes that play many different roles in zebrafish embryogenesis. However, whilst additional roles for zebrafish *hdac1* have also been described in Wnt signalling, in liver and pancreas development, and in the repression of *foxd3* downstream of *mitfa* in specification of neural-crest-derived melanoblasts [31,32,34,35], it is currently unknown whether Atrophin-2 is a component of these particular mechanisms.

In mammals, the developmental functions of NuRD have been deduced from the phenotypes of mouse embryos and ES cells lacking *Mbd3* function. *Mbd3* is essential for early embryogenesis and in culture, *Mbd3* mutant blastocysts fail to proliferate [36^•^]. However, *Mbd3* mutant ES cells are viable but unable to silence genes expressed at preimplantation stages and undergo lineage commitment [37]. Thus, Mbd3/NuRD renders ES cells competent for lineage commitment. A novel NuRD-related complex, NODE, has recently been described which lacks the Mbd3 and RbAp46 subunits but instead binds to the pluripotency-determining transcription factors Nanog and Oct4 [38^•^]. Intriguingly, unlike the loss of *mbd3* function, the knock-down of NODE subunits in ES cells derepressed markers of lineage commitment and induced differentiation, suggesting that through its association with Nanog and Oct4 NODE functions in opposition to Mbd3-containing NuRD complexes to maintain ES cell pluripotency [38^•^]. An emerging theme from the studies of NuRD and Atrophin functions in *C. elegans*, *Drosophila* and mammalian cells is that these SANT-domain-containing protein complexes promote the states of competence that enable cells to respond appropriately to fate-inducing signals, thereby regulating the balance between maintenance of progenitor identity and commitment to differentiation.

## CoREST complex

**Figure 3:**
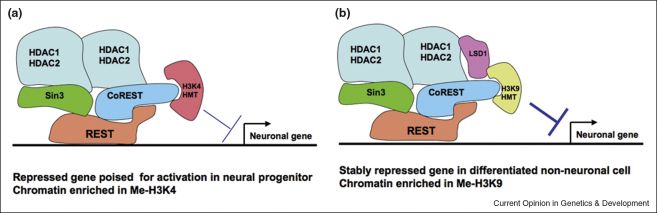
Two distinct modes of REST/CoREST-mediated repression of neuronal target genes in neural progenitor cells and differentiated non-neuronal cells. **(a)** Interaction of the CoREST complex with an H3K4 histone methyltransferase in neural progenitors leads to H3K4 methylation of deacetylated histones, thus poising the associated gene for transcriptional activation at the point of commitment to a neuronal fate [39^•^]. **(b)** Interaction of the CoREST complex with an H3K4-specific histone lysine demethylase (LSD1) coupled with an H3K9-specific histone methyltransferase facilitates the conversion of a silent, poised locus to a stably repressed locus, thus precluding transcriptional activation [40^•^].

In mammalian ES cells, neural progenitors and differentiated non-neuronal cells, the HDAC1/2-containing CoREST complex is recruited by the REST zinc finger protein to RE1 target sites in the promoters of neuronal genes, where it represses transcription [39^•^]. In this complex, DNA-bound REST interacts with Sin3 and CoREST, each of which bind HDAC1/HDAC2 to repress transcription. Like the MTA components of NuRD, the SANT domains of CoREST stimulate HDAC1/HDAC2 activities. In ES and neural stem cells, the HDAC1/HDAC2/CoREST complex also recruits an H3K4 methyltransferase to the RE1 sites of target genes, which methylates H3K4 residues and poises the locus for activation upon commitment to a neuronal fate [39^•^] (Figure 3a). By contrast, in terminally differentiating non-neuronal cells, HDAC1/2-bound CoREST recruits the H3K9 methyltransferase G9a and the H3K4 demethylase LSD1 to the RE1 sites of target genes, which may render these genes refractory to activation [40^•^] (Figure 3b).

Remarkably, many of the key components of the CoREST complex were identified by genetic analysis in *C. elegans* as the repressors of the presenilin gene *hop-1*. Thus, the blockade of Notch pathway activity in the germ-line, by mutation of the *sel-12* presenilin gene, was rescued by mutations in *spr-1/CoREST*, *spr-3/REST*, *spr-4/REST* or *spr-5/LSD1*, each of which independently derepressed the expression of *hop-1* [41,42].

## Interactions of Class I HDACs with CSL and TCF complexes

Both the Notch and the Wnt signalling pathways can activate target genes by antagonising the functions of bespoke HDAC-containing complexes that are tailored to fit the functions of signal-interpreting DNA-binding proteins. Notch pathway activity is transmitted to target genes by binding of the activated Notch intracellular domain (NICD) to the CBF1/Suppressor of Hairless/LAG-1 (CSL) DNA-binding protein. In the absence of NICD, CSL functions as a repressor of Notch targets and interacts with co-repressor complexes that include Hairless, Groucho, SHARP/Spen, CtBP and SMRT, many of which interact directly with Class I HDACs [43]. Hairless-dependent activities of CtBP and Groucho function in the *Drosophila* wing imaginal disc to repress transcription of Notch targets such as *E(spl)mα* and *vestigial* [44,45]. Like CtBP and Groucho, the SHARP/Spen co-repressor also binds directly to Class I HDACs [46]. Interestingly, Spen both antagonises Notch activity and potentiates EGF/Ras/RTK signalling during the development of the *Drosophila* eye [47], but the molecular mechanism of this action is not known. In zebrafish *hdac1* mutants, the Notch target *her6 is* expressed in the CNS independently of a requirement for Notch signalling [26], but whether this mutation also causes altered EGF/Ras/RTK signalling is unclear. Similarly, the transcriptional status of Wnt target genes is determined by the balance of β-catenin/co-activator and HDAC/Groucho co-repressor activities that are associated with the TCF proteins bound to cognate *cis*-regulatory elements [48]. The recent identification of mutations in the NuRD component and zinc finger protein gene *p66*, which modify Wnt signalling in the *Drosophila* wing, provides support for the idea that TCF also mediates NuRD recruitment to target genes [49], although it remains unclear as to whether such a recruitment requires Groucho.

## NCoR/SMRT complexes

The SANT-domain-containing co-repressors SMRT, NCoR and SMRTER interact with Class I HDACs in complexes that are tethered to DNA by transcription factors such as the Notch pathway component CSL and nuclear receptors [43,50]. In the mouse, SMRT and NCoR maintain multipotent neural progenitors and inhibit their differentiation into neurons and astrocytes by a mechanism that involves the repression of an H3K27 histone demethylase [51]. In *Drosophila*, SMRTER interacts with the β-propeller protein Ebi/TBL1, which binds to HDAC3 and deacetylates histones associated with Snail target genes, leading to their transcriptional repression in the embryonic mesoderm [52]. The SMRTER–Ebi complex also acts in association with CSL in the *Drosophila* eye imaginal disc, where, intriguingly, it antagonises Notch-mediated activation of *charlatan*, which encodes a homologue of the REST zinc finger protein [53]. In the wing imaginal disc, by contrast, HDAC3 is required for tissue growth and apoptosis suppression [54], which is reminiscent of the recently described function for zebrafish *hdac3* in promoting liver growth [34].

## Concluding remarks

Class I HDACs play a rich variety of roles in many developmental processes. The breadth of this functional diversity is reflected in the examples discussed in this review:

1. As components of the Sin3 complex, Class I HDACs stabilise positional identity by repressing segmentation and homeotic genes.
2. As parts of a Sin3 complex that interacts with BMP-regulated Smads1/4, Class I HDACs promote BMP-induced transcriptional repression, thus attenuating transcription activated by positive effectors of BMP signalling.
3. Class I HDACs can repress target genes in order to poise them for activation by a signal-induced transcription factor. In the Notch pathway, the activation of target genes by CSL is rendered Notch-dependent by co-repressor complexes that can include Class I HDACs. In *C. elegans*, mammalian cells and *Drosophila*, HDAC-containing complexes also confer repressive functions to the LIN-1/ETS/Yan transcription factors that are bound to target genes, and their actions are antagonised by signalling input from the EGF/Ras/RTK pathway.
4. As a subunit of NuRD, HDAC1 is a component of the transcriptional repression machinery whose sumoylation confers a repressive function to LIN-1/ETS, suggesting that signalling inputs via SUMO could modulate the co-repressor activity of NuRD.
5. Class I HDACs maintain pluripotency and promote lineage commitment as components of NuRD and NODE. Developmental decision-making in the early mammalian embryo may thus be achieved by altering the balance between the activities of these two deacetylase complexes.
6. Through interactions with CoREST, HDAC1/HDAC2 repress neuronal genes in neural precursors and differentiated non-neuronal cells. Additional interactions with distinct histone methyltransferases and histone demethylases may allow the CoREST complex to either poise target genes for activation in neural precursors, or stably repress them in differentiated non-neuronal cells.
7. The NCoR/SMRT/SMRTER co-repressors bind to Class I HDACs and in mammals and *Drosophila* these complexes regulate commitment and differentiation of neural progenitors.

Ongoing investigation of the molecular mechanisms of Class I HDAC function will provide further insights into how these proteins create the context for interpreting developmental signals and thus help to shape the epigenetic landscape within which developmental decisions are taken.

## References and recommended reading

Papers of particular interest, published within the period of review, have been highlighted as:

• of special interest
•• of outstanding interest

## Figures and Tables

**Table 1:** Conservation of Sin3, NuRD, CoREST and NCoR/SMRT components in the Metazoa.

Complex	*C. elegans*	*D. melanogaster*	Vertebrates
Sin3	HDA-1	RPD3	HDAC1, HDAC2
	RBA-1, LIN-53	p55	RbAp46, RbAp48
	SIN-3	Sin3	Sin3A
	MAB-21	*mab-21*	mab21L1, mab21L2
		Sds3	Sds3, BRMS1
			RBP1
	SAP18	*Bin1*	SAP18
			SAP30
			ING1/2

Mi2/NuRD	HDA-1	RPD3	HDAC1, HDAC2
	RBA-1, LIN-53	p55	RbAp46, RbAp48
	LET-418, CHD-3	Mi-2	Mi-2α, Mi-2β
		Atrophin?	MTA1, MTA-2, Atrophin-2?
		MBD2/3	MBD3
		p66	p66α, p66β
	MEP-1	*CG1244*	

CoREST	HDA-1	RPD3	HDAC1, HDAC2
	SPR-1	CoREST	CoREST
	SPR-5	Hdm	LSD1
	SPR-3, SPR-4	REST	
	*DIN-1*	Spen	SHARP
			BHC80
			SIN3

SMRT/NCoR		HDAC3	HDAC3
		SMRTER	SMRT/NCoR
		Ebi	TBL1, TBL1R
			GPS2
			JMJD2A

Putative orthologues without functional data are indicated in italics.
